# Outcome in Patients with Isolated Moderate to Severe Traumatic Brain Injury

**DOI:** 10.1155/2018/3769418

**Published:** 2018-09-23

**Authors:** D. Jochems, K. J. P. van Wessem, R. M. Houwert, H. B. Brouwers, J. W. Dankbaar, M. A. van Es, M. Geurts, A. J. C. Slooter, L. P. H. Leenen

**Affiliations:** ^1^Department of Trauma, University Medical Center Utrecht, Utrecht University, Heidelberglaan 100, 3585 GA Utrecht, Netherlands; ^2^Department of Neurosurgery, University Medical Center Utrecht, Utrecht University, Heidelberglaan 100, 3585 GA Utrecht, Netherlands; ^3^Department of Radiology, University Medical Center Utrecht, Utrecht University, Heidelberglaan 100, 3585 GA Utrecht, Netherlands; ^4^Department of Neurology, University Medical Center Utrecht, Utrecht University, Heidelberglaan 100, 3585 GA Utrecht, Netherlands; ^5^Department of Intensive Care, University Medical Center Utrecht, Utrecht University, Heidelberglaan 100, 3585 GA Utrecht, Netherlands

## Abstract

**Introduction:**

Traumatic brain injury (TBI) remains a major cause of death. Withdrawal of life-sustaining treatment (WLST) can be initiated if there is little anticipated chance of recovery to an acceptable quality of life. The aim of this study was firstly to investigate WLST rates in patients with moderate to severe isolated TBI and secondly to assess outcome data in the survivor group.

**Material and Methods:**

A retrospective cohort study was performed. Patients aged ≥ 18 years with moderate or severe isolated TBI admitted to the ICU of a single academic hospital between 2011 and 2015 were included. Exclusion criteria were isolated spinal cord injury and referrals to and from other hospitals. Gathered data included demographics, mortality, cause of death, WLST, and Glasgow Outcome Scale (GOS) score after three months. Good functional outcome was defined as GOS > 3.

**Results:**

Of 367 patients, 179 patients were included after applying inclusion and exclusion criteria. 55 died during admission (33%), of whom 45 (82%) after WLST. Patients undergoing WLST were older, had worse neurological performance at presentation, and had more radiological abnormalities than patients without WLST. The decision to withdraw life-sustaining treatment was made on the day of admission in 40% of patients. In 33% of these patients, this decision was made while the patient was in the Emergency Department. 71% of survivors had a good functional outcome after three months. No patient left hospital with an unresponsive wakefulness syndrome (UWS) or suffered from UWS after three months. One patient died within three months of discharge.

**Conclusion:**

In-hospital mortality in isolated brain injured patients was 33%. The vast majority died after a decision to withdraw life-sustaining treatment. None of the patients were discharged with an unresponsive wakefulness syndrome.

## 1. Introduction

In 2010, over 56,000 deaths in the USA and 57,000 in the European Union were related to traumatic brain injury (TBI) [1–3]. Furthermore, TBI is the main cause of death in severely injured trauma patients, contributing to 30% of the deaths caused by trauma [1, 2, 4]. Interestingly, mortality rates differ greatly between level-I trauma centers across the world [4].

TBI not only causes mortality, but can also lead to severe functional impairment. Unresponsive wakefulness syndrome (UWS) is a dreaded outcome, in which the patient does not demonstrate any sign of consciousness [5]. Withdrawal of life-sustaining treatment (withdrawal of treatment; WLST) can be initiated when treatment is considered medically futile, in cases where there is negligible chance of recovery to an acceptable quality of life [1, 5, 6].

The Ethicus study [7] investigated end-of-life practices in various ICUs across Europe. Differences in practices between these hospitals included a higher WLST rate in Northern and Central European countries, when compared to countries in Southern Europe. Furthermore, the length of ICU stay before the first treatment limiting decision was significantly shorter in Northern Europe than in the rest of the continent. Amongst patients with acute conditions, neurological disease was the most common motive for treatment limitations [7]. Moreover, a retrospective study in a Dutch ICU found that this was true for WLST as well [8]. However, few studies have published WLST rates, especially not in combination with neurological or functional outcome data.

Therefore, the aim of this study was to investigate WLST rates in patients with moderate to severe isolated TBI and to assess outcome data of the survivors.

## 2. Material and Methods

A local institutional review board (IRB) waiver was formally obtained.

### 2.1. Study Design and Study Population

A retrospective cohort study was conducted including all consecutive patients who sustained isolated moderate or severe traumatic brain injury and were admitted to ICU of the University Medical Center Utrecht (UMCU, a level-1 trauma center) between 2011 and 2015. Isolated moderate or severe brain injury was defined as an Abbreviated Injury Score head & neck (AIShead) of more than three and no significant injury in other regions (defined as AIS of more than two). Patients under 18 years of age, patients with isolated spinal injury without TBI, and referrals to and from other hospitals were excluded. If first CT head showed only subdural and/or parenchymal hemorrhage, patients' records were checked and patients were excluded from analysis, if there was any doubt on whether the brain injury was the consequence or the cause of trauma.

Patients who passed away without WLST were only analyzed for cause of death. This decision was based on the hypothesis that this excluded group will be relatively small, and our main interest was in WLST.

### 2.2. Clinical Variables

Data were collected from medical records and the local trauma database. This database includes several baseline characteristics such as age, sex, ISS, and the AIS of the head region. The trauma mechanism was collected from the medical records. Collected variables included: the Glasgow Coma Scale (GCS) as assessed by the neurologist during the primary survey; pupillary light reflexes and corneal reflexes during primary survey; the need for sedation before arrival or during the stay in the Emergency Department (ED), the concurrent use of a low molecular weight heparin (LMWH); and coumarin or a novel oral anticoagulant (NOAC).

The Charlson Comorbidity Index score was calculated for every patient. This is a widely used score for comorbidity, which comprises 22 comorbidities and each is assigned a weight, according to its impact on the prognosis of the patient [9, 10].

### 2.3. Imaging Variables

In each patient, a noncontrast CT of the head was acquired within 30 minutes after arrival to ED. An experienced neuroradiologist, blinded to the outcome data, revaluated the CT in every patient for the presence of epidural, subdural and/or subarachnoid hemorrhage, compression of the basal cisterns, and midline shift retrospectively.

### 2.4. Outcome Data

Cause of death and WLST data were collected from the medical records. For patients who received WLST, length of stay in ICU was noted. Functional outcome data, measured by the Glasgow Outcome Scale (GOS), were collected at three months (+one month) from records of outpatient clinic visits or correspondence from a neurological rehabilitation center. In case of missing data at three months, the first available GOS was used. If this was before three months' time, it was assessed with the three-month follow-up data, since further deterioration was not expected. If follow-up data were only available after the four-month mark, they were separately analyzed. The GOS allows for objective assessment of the recovery of patients with brain damage in five categories [11]. Good functional outcome was defined as GOS > 3.

### 2.5. Statistical Analysis

All statistical analyses were performed using IBM SPSS Statistics, version 21.0.0 (Armonk, NY, USA). Group differences between survivors and patients who died due to WLST were calculated using a Mann–Whitney U test in case of continuous, nonnormally distributed, variables. In case of a different shape of distributions in each group, mean ranks were compared for analysis of significant differences between groups, and medians were only shown. Differences in distribution of categorical or ordinal variables between groups were calculated with the chi-square test of homogeneity. Fisher's exact test instead of a chi-square test was used if the expected cell count was less than five. Statistical significance was defined as *p*<0.05.

## 3. Results

### 3.1. Study Population

**Figure 1:**
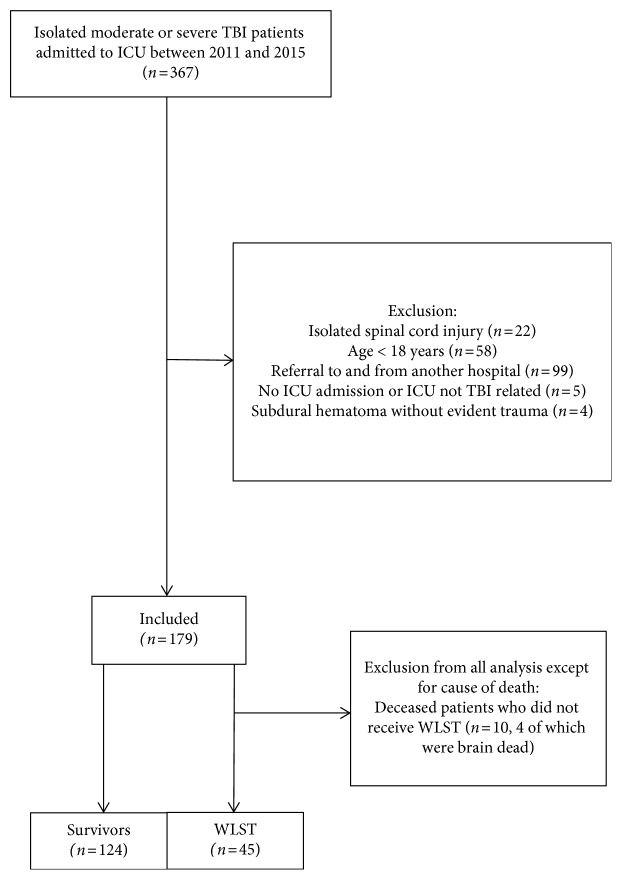
Flowchart of inclusion process. TBI: traumatic brain injury; WLST: withdrawal of life-sustaining treatment.

The search in the trauma registry generated a total of 367 patients with isolated moderate or severe TBI admitted between 2011 and 2015 to the ICU. After applying our exclusion criteria, 179 patients were included in this study (Figure 1). Of these patients, 55 (33%) died during hospitalization (Table 1). The median age at time of the trauma was 57, the median AIS head was four, and the median ISS was 20. Women accounted for 37% (*n*=62) of the patients (Table 1).

Patients for whom WLST was initiated were significantly older and had a median AIShead of five, whereas the AIShead of the non-WLST patients was four (*p*<0.001). Use of coumarins, NOACs, and LMWH was more frequent in WLST patients (*p*=0.043). The difference in mean rank of the Charlson Comorbidity Index was not statistically significant. GCS scores in ED were higher amongst those who did not receive WLST (*p*=0.030). The absence of brainstem reflexes was more common in WLST patients (both *p*<0.01, Table 1). Furthermore, WLST patients were more often sedated before completion of the primary survey than non-WLST patients (*p*=0.005). Subdural hemorrhage, compression of the basal cisterns, and midline shift on the initial CT head were more common in the group of patients who had WLST (all *p*<0.05, Table 1).

### 3.2. Mortality, Surgical Intervention, Complications, and Neurological Outcome

Forty five patients (82%) died following the decision to withdraw life-sustaining treatment. The decision to withdraw life-sustaining treatment was made on the day of admission in 18 cases (40%). In 33% (*n*=6) of those patients, this decision was made whilst the patient was in ED.

10 (22%) of the WLST patients and 13 (10%) of the non-WLST patients received an ICP meter (*p*=0.049). The amount of patients who received neurosurgical decompression during admission did not differ between the non-WLST and WLST group (*p*=0.912) (Table 2).

Median length of stay in ICU before the decision to withdraw life-sustaining treatment was made for patients who received WLST after the first day was 4 days. Of these 27 patients, 12 (44%) suffered from a systemic complication at some point during their admission. In 50% (*n*=6) of these patients, this complication was solely pneumonia (Table 3).

In-hospital mortality as a result of complications occurred in six patients (11%). In two patients, these complications were cardiovascular: These patients died due to a cardiac arrest. Two patients died due to respiratory insufficiency, and one due to a fever in combination with the TBI. One patient had a fever, complicated by respiratory insufficiency, anuria, and diarrhea. This patient had several comorbidities. Four patients (7%) progressed to death by neurological criteria (Figure 1).

None of our patients were discharged to a hospice, since death was expected to follow relatively quickly after the decision to withdraw life-sustaining care.

71% (*n*=78) of the patients with a three-month or later follow-up scored ≥ 4 on the GOS at three months (Table 4). No patient left the hospital with an unresponsive wakefulness syndrome or suffered from UWS after three months. Median GCS on the day of discharge was 15 (IQR: 0). One patient died within three months of discharge. Data concerning GOS were missing in 25% of survivors (Table 4).

## 4. Discussion

We have performed a single-center retrospective analysis on mortality rates, causes of death, WLST, and neurological outcome in patients who were admitted to the ICU with isolated moderate or severe TBI. The mortality rate was 33%, which is comparable to that found in other developed countries (30–40%) [1, 5, 12, 13]. The vast majority of patients died after a decision to withdraw life-sustaining treatment.

There are only four studies that have published rates of WLST in this group of patients. In-hospital mortality rates amongst patients with moderate to severe TBI varied between 10.8% and 44.1%, whilst the WLST rates ranged between 45.0% and 86.6% in these studies: likely due to geographical and cultural differences [1, 5, 14, 15]. Verkade et al. [8] looked at WLST rates in a Dutch ICU. They found that WLST preceded death in 95% of patients who passed away due to irreversible catastrophic cerebral damage [8].

Our WLST rates are at the higher end of the spectrum, when compared to the aforementioned studies; however, they are in range with the earlier published Dutch data [8]. We hypothesize that this may be partly due to cultural differences such as a smaller role of religion in the decision-making [6, 7]. Furthermore, we speculate that people in the Netherlands find quality of life extremely important and therefore might feel that life with UWS has no quality.

Patient wishes were always taken into account. If medical practitioners believe there is no chance of a decent outcome, they will inform the family that medical treatment would be futile. There are no cases in our database where families have doubted or opposed this statement. Unfortunately, due to the retrospective nature of our study, we are not able to trace preexistent patient documents, which might have influenced the decision.

WLST can be appropriate after severe traumatic brain injury to prevent a patient from staying alive at the cost of being left in a state of disability that might be against his or her wishes. However, WLST should not deny patients their chance of a good recovery. Numerous studies have identified several factors with a predictive relationship with outcome after TBI. So far, no model has proven to be perfect, but two widely used prognostication models are the IMPACT score and the CRASH score [20–22]. The risk that WLST may lead to self-fulfilling prophecies, when the prognostic model confirms itself due to physicians basing the decision to WLST on the factors present in this model, has previously been acknowledged for patients with various types of acute brain injury [16–18].

In our study, the decision to withdraw life-sustaining treatment was made in the very acute stage of the disease. Our findings are similar to those of Turgeon et al. [5] where 45.6% of patients who died with WLST did so within the first three days. There is a possibility that patients might have shown clinical improvement if the decision to WLST would have been postponed. In some cases, the decision to WLST has been made when the patient was sedated. The neurological state of these patients has therefore not been assessed. We believe the decision to not discontinue sedation is based on the facts that some patients are clinically not well enough to discontinue sedation or their CT head shows unsalvageable brain damage.

The Neurocritical Care Society therefore suggests delaying withdrawal of treatment and treatment limitations for at least 72 hours in cases of devastating brain injury to give the patient the chance to recover and reduce the risk of prematurely forgoing treatments that could provide clinical benefit [19]. Even though these guidelines were not written for TBI specifically, this raises the concern that the decision to withdraw treatment was made too early in some of the patients in this study.

In addition, the amount of patients who received ICP monitoring was relatively low, when compared to other studies [1, 14]. Even though we have not formally investigated this, we believe that, in line with hospital practice, patients who did not receive an ICP and/or neurosurgical operation were either considered to have a relatively minor injury or unsalvageable catastrophic cerebral damage.

This study has several limitations. Firstly, due to the retrospective nature of this study, we encountered several missing data. One example is the agent and dosage used to sedate the patient. Therefore, we were only able to tell whether the patient was sedated before completion of the primary survey and not if and how this could have affected prognosis. The most important of missing data is that the GOS was not available for all of our patients. Furthermore, at the three-month mark, many patients were still in rehabilitation clinics, but expected to be able to return to an independent life. As such, there is a broad range of neurological outcomes amongst those with a GOS of three, ranging from patients requiring a tracheostomy to those who are on the verge of discharge from the rehabilitation clinic. Patients are not likely to have made their full recovery yet at three months; however, most follow-up data were available until three months. Therefore, future research including a longer follow-up period of these patients is necessary to determine the definite neurological outcome. Using the eight-point GOS scale (the extended GOS) can also specify functional outcome even more and has been recommended in the literature [23]. Unfortunately, a retrospective analysis makes filling out the eight-point scale too difficult; therefore, the five-point scale was considered the more appropriate option, hoping this would prevent misclassification and limit missing data. Furthermore, a comparison of WLST rates between several trauma centers is warranted to establish the exact influence of WLST on mortality and outcome data. In addition, as of today, there is no standard protocol regarding WLST decisions. The decision to withdraw treatment is always taken by the treating physicians including trauma surgeon, neurosurgeon/neurologist, and intensivist and needs to be unanimous before treatment is withdrawn. The lack of standardized documentation of the considerations leading to this decision and therefore lack of analyzed data regarding this subject is a limitation of this study. Finally, we would like to propose a study that investigates the process of, and influences on, the decision to withdraw care.

## 5. Conclusion

The vast majority of in-hospital deaths after moderate or severe TBI occur following a decision to withdraw life-sustaining treatments. Functional outcome of TBI survivors is generally good.

## Figures and Tables

**Table 1:** Baseline characteristics and mortality.

Variable	All patients (*n*=169)	WLST (*n*=45)	Non-WLST (*n*=124)	*P* value
Mortality, *n* (%)^*∗*^	55 (33)	100 (45)	0	
Median age in years (IQR)	57 (32.5)	67 (22)	54 (35.25)	
Mean rank age	n/a	114.56	74.27	<0.001
Median ISS (IQR)	20 (9)	25 (9)	20 (9)	0.01
Median AIShead (IQR)	4 (1)	5 (1)	4 (1)	
Mean rank AIShead	n/a	108.47	76.48	<0.001
Female, *n* (%)	62 (37)	16 (36)	46 (37)	0.854
Trauma mechanism, *n* (%)				0.061
Fall stairs or height	53 (32)	19 (44)	34 (28)	
Fall low height/collaps or nos	15 (24)	8 (19)	16 (13)	
Traffic accident: two wheels	60 (36)	11 (26)	49 (41)	
Traffic accident: car	12 (7)	1 (2)	11 (10)	
Traffic accident: pedestrian	4 (2)	1 (2)	3 (3)	
Hit by subject	6 (4)	0 (0)	6 (5)	
Penetrating injury	3 (2)	2 (5)	1 (1)	
Hanging	2 (1)	1 (2)	1 (1)	
Missing	5(3)	2 (4)	3 (2)	
Charlson Comorbidity Index (IQR)				
Median		0 (1)	0 (1)	
Mean rank		88.37	81.23	0.302
Anticoagulant users, *n* (%)				
None or platelet aggregation inhibitors		32 (82)	116 (94)	0.043
Coumarins/heparines/NOAC		7 (18)	7 (6)	
Missing		6 (13)	1 (1)	
GCS in ED (IQR)				
Median		7 (8)	8.5 (5)	
Mean rank		47.54	64.51	0.030
Sedated, *n* (%)		20 (44)	28 (23)	
Motor score in ED (IQR)				
Median		5 (4)	5 (1)	0.009
Missing, *n* (%)		20 (44)	28 (23)	
Pupil reflexes in ED, *n* (%)				
None or one eye		21 (50)	17 (15)	<0.001
Both eyes		21 (50)	96 (85)	
Missing		3 (7)	11 (9)	
Corneal reflexes in ED, *n* (%)				
None or one eye		11 (58)	4 (17)	0.009
Both eyes		8 (42)	20 (83)	
Missing		26 (58)	100 (81)	
Sedation, *n* (%)		20 (44)	28 (23)	0.005
Signs on first CT scan, *n* (%)				
Epidural hemorrhage		17 (38)	42 (34)	0.716
Subdural hemorrhage		43 (96)	93 (75)	0.004
Subarachnoidal hemorrhage		39 (87)	91 (74)	0.097
Compression basal cisterns		34 (76)	52 (42)	<0.001
Midline shift		25 (56)	33 (27)	0.001

WLST: withdrawal of life-sustaining treatment; AIShead: Abbreviated Injury Score of the head region. ^*∗*^10 patients who died due to other causes than WLST are included in this analysis.

**Table 2:** Neurosurgery and ICP meter.

Neurosurgery variables	WLST, *n*=45	Non-WLST, *n*=124	*P* value
Received ICP meter, *n* (%)	10 (22)	13 (10)	0.049
Underwent neurosurgical decompression, *n* (%)	17 (38)	48 (39)	0.912

WLST: withdrawal of life-sustaining treatment; ICP: intracranial pressure.

**Table 3:** ICU parameters for patients who did not receive WLST on the first day.

ICU variables	WLST, *n*=27	Non-WLST, *n*=124
Median length of stay in ICU in days (IQR)	4 (5)	3 (5)
Median length of stay in hospital in days (IQR)	n/a	17.5 (21.75)
WLST following systemic complications	*n*=12 (44%)	n/a

WLST: withdrawal of life-sustaining treatment; n/a: not applicable.

**Table 4:** GOS score and destination after discharge of the survivor group. The GOS score at three to four months or less of patients who were alive at discharge, expressed in percentage per GOS score.

Glasgow Outcome Scale	Patients assessed at three months, *n*=124 (%)	Patients assessed after three months, *n*=23 (%)	All assessed patients, *n*=124 (%)	Discharged to	Patients, *n*=124 (%)
1	1 (1)	0 (0)	1 (0)	Home	50 (40)
2	0 (0)	0 (0)	0 (0)	Rehab	69 (56)
3	31 (31)	0 (0)	31 (28)	Home against medical advice	3 (2)
4	52 (51)	5 (56)	57 (52)		
5	17 (17)	4 (44)	21 (19)	Psychiatry ward	2 (2)
Missing	23 (19)	14 (61)	14 (11)		0 (0)

The individual values are rounded to the nearest percent and may not total 100%. Score 1 is defined as death and score 2 is unresponsive wakefulness syndrome. Score 3 is defined as severe injury with permanent need for help with daily living, and score 4 is moderate disability; no need for assistance in everyday life, employment is possible, but may require special equipment. When a patient scores a 5, he or she has only minor deficits in the physical, social, or psychological domain [16].

## Data Availability

The data from the medical records used to support the findings of this study are restricted by the Medisch Ethische Toetsingscommissie (Medical Ethical Committee) in order to protect patient privacy. Pseudonymized data are available from the corresponding author for researchers who meet the criteria for access to confidential data.
